# Dermoscopy Training Effect on Diagnostic Accuracy of Skin Lesions in Canadian Family Medicine Physicians Using the Triage Amalgamated Dermoscopic Algorithm

**DOI:** 10.5826/dpc.1002a35

**Published:** 2020-04-03

**Authors:** Elizabeth A. Sawyers, Donald T. Wigle, Ashfaq A. Marghoob, Andreas Blum

**Affiliations:** 1 Community Family Practice, Ottawa, Canada; 2 Medical Epidemiology, Ottawa, Canada; 3 Dermatology, Memorial Sloan Kettering Cancer Center, New York, NY, USA; 4 Dermatology, Eberhard Karls Universitat, Tubingen, Germany

**Keywords:** dermoscopy, family/general practice, skin cancer, medical education, Triage Amalgamated Dermoscopic Algorithm

## Abstract

**Background:**

Accurate identification of cutaneous lesions is an essential skill for family medicine physicians (FMPs). Studies show significant improvement in skin cancer detection with dermoscopy use. Frontline FMPs are an ideal target group for dermoscopy training. The 3-step Triage Amalgamated Dermoscopic Algorithm (TADA) facilitates high sensitivity and specificity for pigmented and nonpigmented skin lesions. Step I requires unequivocal identification of dermoscopic features for 1 of 3 benign skin lesions: angioma, dermatofibroma, or seborrheic keratosis. If absent, steps II and III are applied assessing for features of architectural disorder and malignancies with organized, symmetric patterns, respectively.

**Objective:**

To assess FMPs’ diagnostic accuracy of benign and malignant skin lesions before and after training in TADA step I.

**Methods:**

In this repeated-measures observational study, 33 dermoscopy-naive FMPs attending an introductory dermoscopy workshop each assessed gross and corresponding dermoscopic photographic images of 50 pigmented and nonpigmented skin lesions (23 benign, 27 malignant) for features of TADA step I lesions before and after training. Analyses compared diagnostic accuracy in relation to training and baseline physician characteristics.

**Results:**

Diagnostic accuracy improved from 76.4% to 90.8% (P < 0.001) and from 85.0% to 90.0% (P = 0.01), respectively, for all lesions and for all TADA I lesions. Female sex was significant as a predictor of individual posttraining performance (all lesions combined, P = 0.02).

**Conclusions:**

Results show significant improvement in diagnostic accuracies for benign and malignant skin lesions with introductory dermoscopy training using TADA step I. This will reduce unnecessary benign lesion excision and enhance referral sensitivity, conserving specialist resources.

## Introduction

Skin cancer is the most common of all Canadian cancers, with more than 80,000 cases diagnosed yearly [1]. In 2017, 7,200 of these were melanomas, the most lethal of subtypes [1]. While incidence and mortality rates persistently climb, Canadians have difficulty accessing dermatological specialty care [2,3]. Frontline family medicine physicians (FMPs) are ideally positioned to identify cutaneous lesions with malignant potential.

Dermoscopy is an inexpensive, noninvasive tool shown to significantly improve diagnostic accuracies of benign and malignant skin lesions compared with naked eye examination among FMPs and dermatologists [4–6].

The complex scoring systems and/or narrow focus of previously validated dermoscopic algorithms make them impractical for primary care setting integration [7–9]. Triage algorithms, however, eliminate requirements for specific diagnoses, asking users to distinguish benign from potentially malignant lesions, the latter necessitating biopsy or referral [10,11].

**Figure 1:**
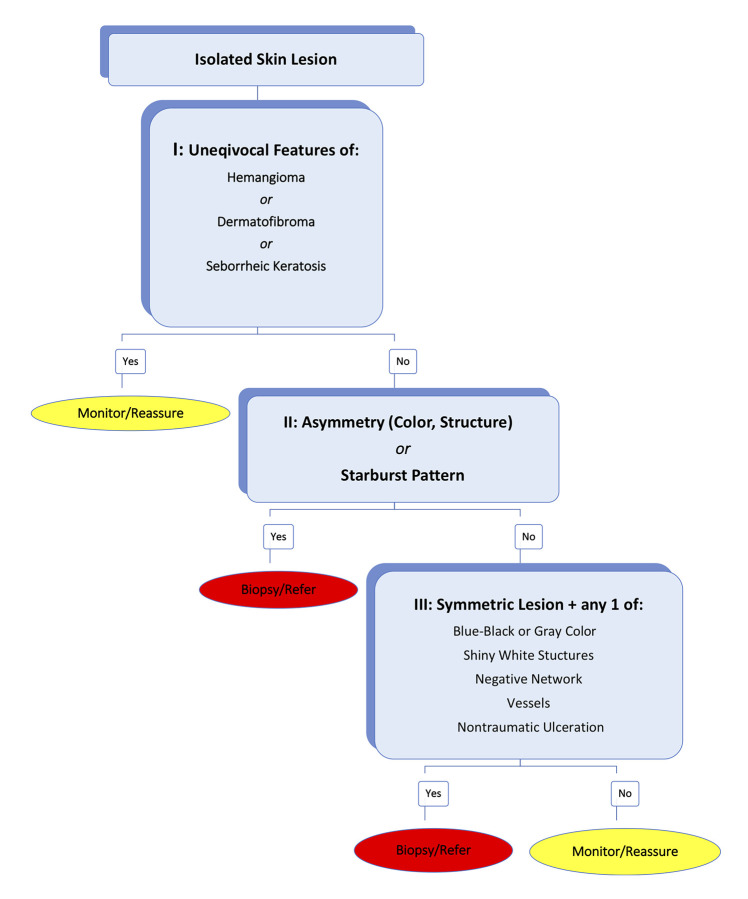
The Triage Amalgamated Dermoscopic Algorithm [10–12].

**Figure 2:**
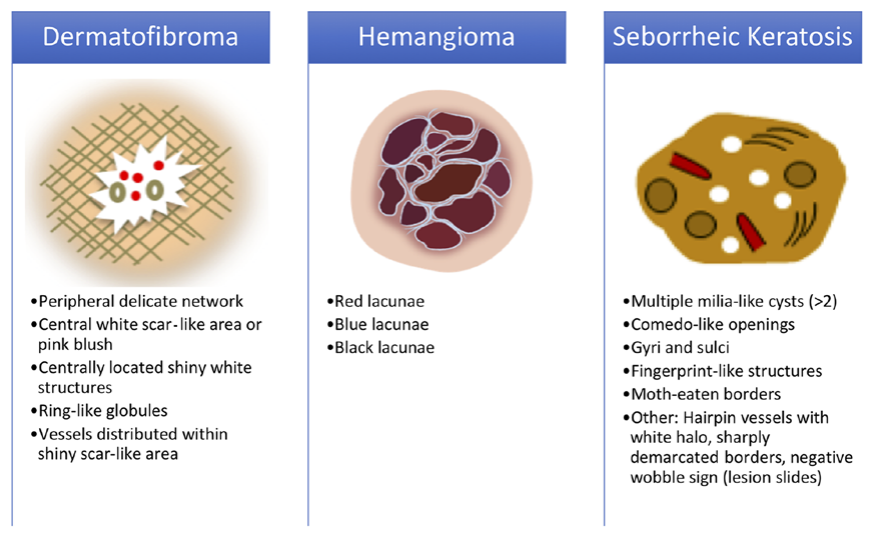
Unequivocal features of skin lesions assessed in the Triage Amalgamated Dermoscopic Algorithm step I [10–12].

The 3-step Triage Amalgamated Dermoscopic Algorithm (TADA), unlike others, allows assessment of pigmented, nonpigmented, benign, and malignant skin lesions including melanomas demonstrating symmetric homogenous patterns [10,11]. Step I looks to unequivocally identify dermoscopic criteria in any 1 of 3 benign lesions (angioma, dermatofibroma, or seborrheic keratosis). If absent, step II is applied, assessing for features of architectural disorder, and similarly, step III for malignancies with organized, symmetric patterns (Figures 1 and 2) [10,11]. TADA requires polarized light dermoscopy use and excludes facial, mucosal, nail unit, and glabrous skin lesion analysis [10,11].

As Canadian dermoscopy training opportunities are sparse for FMPs, our objective was to develop and test introductory educational modules for TADA step I in a group of FMPs.

## Methods

This repeated-measures study conducted in Ottawa, Canada, included 33 dermoscopy-naive FMPs voluntarily responding to a mass mailing invitation to a 3.5-hour introductory dermoscopy workshop without compensation. Incomplete data sets for 2 participants were excluded, leaving 31 for analyses.

Participant characteristics, potential barriers to dermoscopy use, and familiarity with Canadian screening guidelines for diabetes mellitus, hypertension, cancers (breast, cervical, colorectal, prostate, skin), and osteoporosis were collected by questionnaire upon registration. TADA step I teaching modules were written and presented live on Microsoft Power Point for Mac v15.33, 2017 (Microsoft Corporation). Content introduced fundamental dermoscopy principles with emphasis on TADA and review of published dermoscopic findings of the 3 target lesions (dermatofibroma, hemangioma, seborrheic keratosis) [10–12].

Participants were provided a 25-minute session to complete each identical pre- and postteaching quiz of 50 randomly ordered cases projected onto a large screen for participant viewing and assessment, each containing clinical and corresponding dermoscopic images with a brief patient history (age, sex, lesion location, relevant clinical lesion features). Cases included 27 malignant and 23 benign lesions: 7 basal cell carcinomas (5 nonpigmented, 2 pigmented), 4 squamous cell carcinomas (3 in situ, [Bowen disease], 1 invasive; 2 nonpigmented, 2 pigmented), 16 melanomas (9 in situ, 1 nodular, 1 amelanotic, and 5 superficial spreading) with mean Breslow tumor thickness of 1.24 mm, 8 nevi, 5 angiomas, 5 seborrheic keratoses, 4 dermatofibromas, and 1 clear cell acanthoma.

Lesion diagnosis was confirmed based on the presence of unequivocal clinical and/or dermoscopic criteria for those benign and by histopathology for those ambiguous and/or malignant.

Photographic images selected retrospectively from patient records were obtained with a Canon Rebel T1i EOS 500D (Canon Canada, Brampton, ON, Canada) and/or iPhone 6 (Apple) camera attachment using contact polarized dermoscopy (Dermlite DL3N, 3Gen Inc.) with magnification ×10. Four quiz images were provided by authors Andreas Blum (Germany; A.B.), Ashfaq A. Marghoob (USA; A.A.M.), and contributor Darshini Persaude (Canada; D.P.). Presented material was preapproved by expert dermoscopists and authors A.B. and/or A.A.M.

Participants were instructed to choose 1 of 4 possible responses per case in provided quiz booklets to identify lesions possessing the published dermoscopic features of angioma, dermatofibroma, seborrheic keratosis (Figure 2), or none of these. The latter category, in which no dermoscopic features of TADA I lesions were identified, was deemed non-TADA I lesions representing all malignant lesions, nevi, and clear cell acanthoma. Participants’ pre- and postteaching quiz response data for a maximum possible total of 3,100 lesion assessments (31 participants × 50 cases × 2 assessments) were collected and coded numerically for each of the 4 questions (0 = did not answer; 1 = seborrheic keratosis; 2 = hemangioma; 3 = dermatofibroma; 4 = none of the these) and entered into Microsoft Excel for Mac v15.33, 2017 for statistical analysis.

Sensitivity and specificity were defined, respectively, as the percentage of malignancies correctly identified as non-TADA I and the percentage of TADA I lesions correctly identified as such.

Pre- and postteaching accuracy scores for all skin lesions and subgroups were evaluated with a matched-pairs t test using Wizard Pro for Mac [13]. Postteaching accuracy scores for all lesions combined were assessed in relation to 5 independent participant characteristics: age (>50 vs up to 50 years old), sex (M, F), years in practice (>10 vs up to 10 years), medical teaching within their practice (yes vs no), and number of skin cancers diagnosed during the past 6 months (up to 5 vs ≥6) using a t test [13]. All scores were normally distributed except for pre- and postteaching for TADA I lesions and postteaching for melanoma. Given our study sample size over 30, the paired t test is valid for measuring statistical significance [14].

## Results

Table 1 lists participant characteristics. Among these, only female sex was statistically significant as a predictor of postteaching diagnostic accuracy. The mean numbers of correct postteaching diagnoses for all lesions combined among men and women, respectively, were 44.5 and 45.9 (t test, P < 0.001).

**Figure 3:**
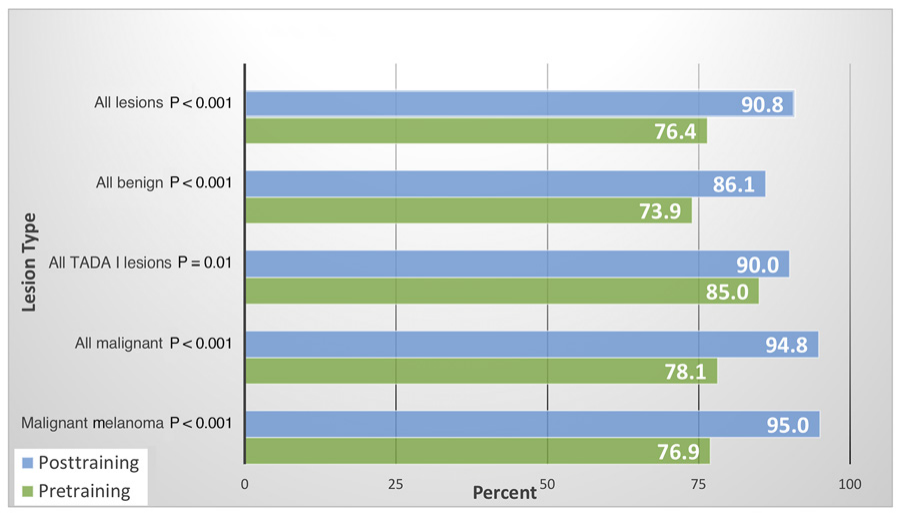
Comparison of percent correct diagnosis by skin lesion group pre- and posttraining by participants using the Triage Amalgamated Dermoscopic Algorithm (TADA) step I.

Table 2 shows correct diagnoses for all skin lesions improved from 76.4% to 90.8% (paired t test, P < 0.001) after teaching (Figure 3). For the TADA I lesions, pre- to postteaching specificity, as defined, increased from 85.0% to 90.0% (paired t test, P = 0.01). Thus the percentage of TADA I lesions unnecessarily biopsied would decrease by one-third from 15% to 10% after training. Sensitivity, as defined, for all malignancies increased from 78.1% to 94.8% (paired t test, P < 0.001) and for malignant melanoma subcategory from 76.9% to 95.0% (paired t test, P < 0.001).

**Figure 4:**
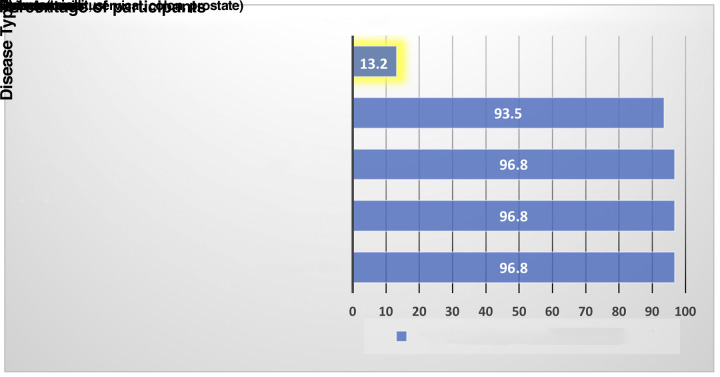
Participant familiarity with Canadian clinical practice guidelines for common diseases seen in primary care.

Perceived barriers to FMPs’ dermoscopy use were identified as lack of awareness regarding dermoscopy (67.7%), training time required to achieve proficiency (61.3%), and lack of accessible training programs (54.8%). More than 90% of participants were familiar with Canadian clinical practice guidelines for diabetes mellitus, hypertension, targeted cancers, and osteoporosis, with just 12.1% aware of those for skin cancer (Figure 4).

## Discussion

Our results demonstrate that introductory dermoscopy training and application of TADA step I significantly improved FMPs’ diagnostic accuracy for both benign and potentially malignant lesions. This addresses the growing need for such training given current escalating strains on Canadian dermatological resources.

The Canadian Skin Patient Alliance reports failing grades in the standard-of-care benchmark for median wait times for any dermatological patient from 5 to 12 weeks from 2001 to 2011 and 23 weeks for 25% of patients in 2011 [15]. In 2011, 55% of melanoma patients had wait times exceeding the 2-week benchmark for assessment [15].

The Royal College of Physicians and Surgeons of Canada suggests a benchmark ratio of 1.5 dermatologists to 100,000 people, which is not achieved in 55% of the provinces and territories, with 18% and 73% having ratios <0.5 and ≤1.5, respectively [15,16]. With 46% of practicing Canadian dermatologists being ≥55 years old and a projected 3.4% annual attrition rate, the 6.2% annual increase in full-time dermatologists required for replenishment is unattainable at the existing 0.8% replacement rate [15,16].

Strategic partnership with Canada’s 43,500 FMPs potentially could improve early skin cancer detection; however, several barriers exist [17]. Dermatological training is underrepresented in medical education, nationally and internationally [18–20]. Large proportions of new American graduates feel inadequately trained to evaluate skin conditions, while 82% of UK graduates report never being exposed to a single type of skin cancer [18].

Designated hours of medical education for Canada’s Dalhousie University 2017 graduating class were 13 for dermatology compared with 107, 78.5, and 69 for neurology, cardiology, and respirology, respectively [18]. A 2017 survey from Canadian medical schools showed 75% of dermatological core educational hours occurred in preclerkship years, with only 3 schools offering compulsory clinical exposure, a decrease from 12 in 1983 [21]. It is not surprising that most melanoma patients have had 1 or more physician visits the year before diagnosis, with only 20% receiving a screening examination [4,22]. Our finding of low familiarity with clinical practice guidelines for skin cancer compared with other illnesses is consistent with other studies illustrating dermatological educational deficits [23,24].

The Canadian Professors of Dermatology 2012 national core curriculum lists skin cancer recognition as an expected competency without mention of dermoscopy training to facilitate this goal [20]. Inadequate dermoscopic training results in diagnostic outcomes that are inferior to those from the naked eye for melanoma [25], but FMPs given even short training had improved clinical and dermoscopic diagnostic sensitivity [26]. A randomized controlled trial showed a 25.1% difference (54.1% vs 79.2%, P = 0.002) in referral sensitivity for lesions of malignant suspicion in FMPs using dermoscopy compared with the naked eye, without compromising specificity [4]. A study using the complete TADA found sensitivity and specificity rates of 94.6% (95% confidence interval = 93.4%–95.7%) and 72.5% (95% confidence interval = 70.1%–74.7%), respectively, after a 1-day training session for physicians, some of whom had previous dermoscopy experience [11].

Our inclusion of only dermoscopy-naive FMPs indicates TADA’s utility in novices [10,11]. Our results outperform those in a randomized controlled trial of FMPs trained with the Three-Point Checklist triage algorithm in identifying lesions of malignant potential (sensitivity 79.2%, specificity 71.8%) [4].

Although studies report that time required for dermoscopy-assisted examinations is a barrier to uptake [27–29], we found no such evidence. A randomized, prospective multicenter analysis concluded that use is not a significant burden, with a median time difference of only 72 seconds (70 vs 142) for total body skin examination with and without dermoscopy [29].

We show potential for impactful clinical risk reduction with the 2-fold decrease in unnecessary benign lesion biopsy rates after training.

TADA I identifies only the 3 targeted benign skin lesions, leaving a potentially malignant group requiring analysis with subsequent steps II and III. Our reported sensitivity and specificity are not equivalent to those obtained from testing the full algorithm. However, we report significant improvements comparing favorably to those published [11]. Improved diagnostic accuracy of step I lesions reduces the lesion pool progressing to steps II and III and the probability of false positives [10,11]. Our results support this with greater changes in diagnostic accuracies realized for malignant vs TADA I lesions, respectively, at 16.7% (P < 0.001) and 5.0% (P = 0.01).

Although participants did not directly examine patients, clinical information, skin lesion, and dermoscopic photographs were provided to simulate realistic clinical encounters [10,11].

Acknowledging the limitation of a small number of self-selected participants, our conservative data assessment used a matched-pairs t test to assess statistical significance of pre- and posttraining diagnostic score changes.

## Conclusions

We show that introductory training of dermoscopy-naive FMPs resulted in significant improvements in diagnostic accuracy of benign and malignant skin lesions, with potential for improved referral sensitivity and reduction in unnecessary benign lesion excisions. Training in TADA steps II and III is necessary to evaluate directly the diagnostic accuracy of non-TADA I lesions and is a planned future endeavor to provide FMPs the required skill set to respond to current and future dermatological clinical demands.

## Figures and Tables

**Table 1:** Participant Characteristics (n = 31)

Characteristic	% (n)
**Age**	
**Up to 50 years**	**48.4% (15)**
20–30 years	12.9% (4)
31–40 years	19.4% (6)
41–50 years	16.1% (5)
**>50 years**	**51.6% (16)**
51–60 years	19.4% (6)
>61 years	32.2% (10)
**Sex**	
Male	35.5% (11)
Female	64.5% (20)
**Years in practice**	
**Up to 10 years**	**38.7% (12)**
<1 year	6.5% (2)
1–3 years	9.7% (3)
>3–5 years	9.7% (3)
>5–10 years	12.9% (4)
**>10 years**	**61.3% (19)**
>10–15 years	3.2% (1)
>15 years	58.1% (18)
**Teaching in practice**	
Yes	41.9% (13)
No	54.8% (17)
Did not answer	3.2% (1)
**Skin cancers diagnosed in past 6 months**	
**Up to 5**	**61.3% (19)**
**6 or more**	**35.5% (11)**
6–10	19.4% (6)
11–15	3.2% (1)
16–20	9.7% (3)
>21	3.2% (1)
**Did not answer**	**3.0% (1)**

**Table 2:** Comparison of Pre- and Posttraining Percent Correct Responses for 1,550 Skin Lesion Assessments by Participants Using the Triage Amalgamated Dermoscopic Algorithm (TADA) Step I

Skin Lesion Subgroup (n)	Percent Correct Diagnoses		P Value
	Pretraining	Posttraining	
All lesions (50)	76.4	90.8	<0.001
All benign (23)	73.9	86.1	<0.001
Angioma (5)	90.0	96.0	0.06
Clear cell acanthoma (1)	60.0	90.0	0.005
Dermatofibroma (4)	90.0	97.5	0.03
Nevi (8)	56.3	77.5	<0.001
Seborrheic keratosis (5)	74.0	78.0	0.50
All TADA I lesions (14)	85.0	90.0	0.01
All malignant (27)	78.1	94.8	<0.001
Basal cell carcinoma (7)	85.7	98.6	<0.001
Bowen disease (3)	66.7	83.3	0.02
Malignant melanoma (16)	76.9	95.0	<0.001
Squamous cell carcinoma (1)	80.0	100.0	0.01

Correct responses were classified as a specific TADA I lesion (angioma, dermatofibroma, or seborrheic keratosis) or “other” if a non-TADA I lesion.
